# Evaluation of the Continuing Education Training “Beratende für Digitale Gesundheitsversorgung” (“Consultant for Digital Healthcare”): Protocol for an Effectiveness Study

**DOI:** 10.2196/57860

**Published:** 2024-09-04

**Authors:** Bernhard Kraft, Thomas Kuscher, Susann Zawatzki, Sebastian Hofstetter, Patrick Jahn

**Affiliations:** 1 Health Service Research Working Group | Acute Care Faculty of Medicine Martin Luther University Halle-Wittenberg Halle (Saale) Germany; 2 Institute for History and Ethics of Medicine Faculty of Medicine Martin Luther University Halle-Wittenberg Halle (Saale) Germany; 3 Dorothea Erxleben Lernzentrum Halle Faculty of Medicine Martin Luther University Halle-Wittenberg Halle (Saale) Germany

**Keywords:** digitization, digital-assistive technologies, nursing and health care professionals, further training program, digital competence, digital healthcare, digital health, effectiveness study, training program, nursing, nursings, health professional, health professionals, transfer of learning, satisfaction, mixed-method, self-assessment, Teaching Analysis Poll, technology, technologies, innovation, exploratory, intervention

## Abstract

**Background:**

The digital transformation in health care requires training nursing and health professionals in the digitally competent use of digital assistive technologies (DAT). The continuing education training “Beratende für digitale Gesundheitsversorgung” (“Consultant for Digital Healthcare”) was developed to fill this gap. The effectiveness of the training program will be assessed in this study.

**Objective:**

The primary objective is to record and measure the participants’ learning success. We will assess whether the previously defined teaching intentions, learning objectives, competencies, and participants’ expectations have been achieved and whether a transfer of learning occurred. The secondary objective is participant satisfaction and feasibility of the training. The tertiary objective is the successful transfer of DAT by participants in their institutions.

**Methods:**

Approximately 65 nursing and health care professionals will participate in the pilot phase of the further training and evaluation process, which is planned in a mixed methods design in a nonsequential manner. The different methods will be combined in the interpretation of the results to achieve a synaptic view of the training program. We plan to conduct pre-post surveys in the form of participant self-assessments about dealing with DAT and content-related knowledge levels. Exploratory individual interviews will also be conducted to build theory, to examine whether and to what extent competence (cognition) has increased, and whether dealing (affect) with DAT has changed. Furthermore, an interim evaluation within the framework of the Teaching Analysis Poll (TAP) will occur. The knowledge thereby gained will be used to revise and adapt the modules for future courses. To assess the transfer success, the participants create a practical project, which is carried out within the training framework, observed by the lecturers, and subsequently evaluated and adapted.

**Results:**

We expect that the learning objectives for the continuing education training will be met. The attendees are expected to increase their level of digital competence in different skills areas: (1) theoretical knowledge, (2) hands-on skills for planning the application and practical use of DAT, (3) reflective skills and applying ethical and legal considerations in their use, (4) applying all that in a structured process of technology implementation within their practical sphere of work.

**Conclusions:**

The aim of this study and appropriate further training program are to educate nursing and health care professionals in the use of DAT, thereby empowering them for a structured change process toward digitally aided care. This focus gives rise to the following research questions: First, how should further training programs be developed, and which focus is appropriate for addressee-appropriate learning goals, course structure, and general curriculum? Second, how should a training program with this specific content and area be evaluated? Third, what are the conditions to offer a continued program?

**International Registered Report Identifier (IRRID):**

PRR1-10.2196/57860

## Introduction

The use of existing and development of new and innovative digital assistive technologies (DAT) offer great potential to meet both current and future challenges in care. DAT is a collective term for systems that are intended to support people in their diverse tasks with the aim of promoting independence and self-sufficiency, expanding one's own abilities, and promoting well-being [1,2]. In addition, it aims to improve quality of life when used in a planned, problem-oriented manner [3,4]. Furthermore, DAT is discussed as a useful support mechanism for caregivers by enabling them to be additional resources for health care provision. When used correctly, DAT creates relief opportunities and strengthens the willingness of family members to provide care [5,6]. In the sense of “on-site” assistance, DAT can thus increase the radius of action of people in their environment. The Organisation for Economic Co-operation and Development's aging-in-place approach is authoritative in this regard [7]. Consequently, health professionals can also benefit from the acquisition of transformative competencies and the participatory acquisition of health-related technical knowledge.

The targeted and user-centered use of digital technologies in the health care sector can lead to improved care and offers countless potential opportunities. Health and care professionals can be supported and relieved in their daily work, and the recipient of health services can experience an increase in the quality of the service thanks to a technically supported offering, thereby improving their own quality of life [8,9].

Of course, there are also ethical, social, and practical challenges concerning the use and implementation of digital technologies in the health care sector that need to be addressed. These are, for example, questions of acceptance, data protection, the corresponding integration into existing work processes, and how they are financed [10-16].

Since digitization in nursing care facilities has been comparatively underdeveloped to date, the sector is about to undergo a profound change [17-19]. Preparing the players involved for this and enabling them to actively shape the transformation and contribute their professional expertise to the development, design, and implementation of new technologies are necessary educational tasks for the success of this process. The necessary competencies should be learned in practical, daily-use scenarios in order for the technologies and new processes to be confidently integrated into nursing practice [20,21].

In order to systematically and broadly familiarize health care and nursing staff with the topic, it is necessary to anchor the teaching of digital competencies in education, training, and continuing education [22]. The need and willingness to learn the practical use of DAT are strong among nursing trainees [23]. Nevertheless, these requirements have so far been insufficiently implemented in the framework curricula of the federal states and in the design of targeted continuing education programs in Germany [23,24].

Comparable continuing education offerings, such as the “Online Campus Pflege” or “Pflege 4.0,” mostly focus on simply teaching digital skills and involve a relatively low number of hours, especially the face-to-face sessions [25-28]. In contrast to these offerings, the continuing education training “Consultant for Digital Healthcare” is characterized by a high level of application orientation, emphasizes ethical and reflective skills transfer, and, finally, is accompanied by and focuses on a practical project in which the implementation of digital technologies is supported in practice. In addition to imparting theoretical knowledge and practical application skills, the training aims to have a direct influence on digital transformation and to demonstrate and accompany the successful theory-practice transfer with the resulting projects.

This research project would like to close the gap and pursue the research question of how digital competencies should be taught in an addressee-appropriate manner in the form of continuing education in order to enable professionals to become sovereign designers and multipliers of the digital transformation in health care and health care delivery. Therefore, the training program “Consultant for Digital Healthcare” shall be evaluated.

## Methods

### Study Design

The study is planned as an exploratory intervention feasibility study with a follow-up survey. Through a mixed methods approach, the qualitative and quantitative survey methods used are combined in a nonsequential design [29,30]. This means that the results of the individual parts should enable a better overall understanding of the research questions raised [30].

The aim of the study is to evaluate whether the continuing education program for “Consultants for Digital Healthcare” empowers nursing and health care professionals to shape the digital transformation, specifically by serving as multipliers for transformation projects in various health care facilities. This is intended to promote the sustainable implementation of digital systems in practice. To achieve this, multipliers require diverse competencies, including not only technical expertise but also skills in guiding, advising, and training colleagues. The multiplier can be broadly considered a change agent. For this reason, digital competence is understood here as a cross-sectional skill that should enable a diverse, multidimensional approach to aspects of digital transformation.

To be able to evaluate the structure, learning objectives, and didactic methods of further education scientifically, they can be examined using a mixed methods design. For this purpose, previously offered events were systematically reviewed through literature research and compared with existing theory (Project Format) on the development of digital competencies [12,17,31,32]. This comparison revealed that corresponding continuing education programs, which understand the conveyance of digital competency as a cross-sectional skill and thus aim for sustainable, connectable development, are underrepresented. Against this background, a blended learning concept was developed based on theoretical and evidence-based considerations that promote learning and practical transfer through action-guided instruction and aim to address the preceding research desideratum. The hypothesis underlying this is that the use and conveyance of specific assessment tools and action guides and the practical design of training situations promote learning transfer.

The level of outcomes reflects the learning and success monitoring of the further education program. We examine whether participants can build digital competencies in handling digital and assistive technologies and transfer them into practice. The structure primarily reflects the macrodidactic framework. This aims to assess whether the resources available and used for the learning situations were appropriate and effective. The process describes the course of the pilot phase and serves to monitor the success of implementation and feasibility of the project. 

The evaluation of learning success will be conducted quantitatively through a self-assessment questionnaire and item questions on participants’ knowledge content. The questions relate to the 2 dimensions of cognition and affect in dealing with digital and assistive technologies, with cognition being the primary dimension reflected. It is assumed that these dimensions function similarly to attitudinal characteristics. The questionnaire was developed along various areas of action-oriented competence development in health care, such as areas of professional, social-communicative, ethical-reflective, and general methodological competence. [33]. By promoting self-efficacy, either through the building of knowledge content or the reduction of barriers to deal with DAT, transfer into practice as well as acceptance in use can be promoted. For the qualitative assessment of the training by the participants, guided interviews are used. The questions were developed using the SPSS (collect, check, subsume, and sort) method [34]. These will be evaluated using qualitative content analysis, according to the method described by Kuckartz and Radiker [35]. Deductively derived categories from theory will be examined using a coding system and checked for fit. Furthermore, the coding allows for the formation of inductive categories to identify further codes, which is intended to promote theory building and examination in the field of digital competencies.

Another tool consists of standardized questionnaires to evaluate specific training situations. These assess various dimensions of the teaching situation, including spatial setup, lecturer skills, and atmosphere.

Additionally, through a practical project conducted by the participants and the associated observation by the lecturers in terms of open observation, the behavioral dimension of the participants dealing with DAT becomes visible and is documented by the lecturers using observation sheets. Further education thus provides the opportunity to assess not only acquired competencies in closed training settings but also directly with the participants and to finally reflect on them.

We planned 3 measurement points for the study. The first measurement point (t1) occurs before direct participation. Here, self-assessment questionnaires and the participants' knowledge of dealing with DAT are recorded. The second measurement point occurs at the halfway mark of the respective conducted modules (t2). At this point, the first revision of the modules is made using the Teaching Analysis Poll (TAP). The third measurement point occurs after the completion of the continuing education (t3). Here, the self-assessment questionnaires or knowledge levels for dealing with DAT are recorded again. Furthermore, at this point, the qualitative individual interviews are conducted. Since participants are not subject to strict scheduling due to the modular structure, there are no scheduled dates but individual measurement points for the participants.

The target group is nursing and health professionals, and the prerequisite for further training is basic vocational training in those professions. With this target group in mind, the training is designed according to the Deutscher Qualifikationsrahmen (German qualification framework) with a requirement level of level 4 [36]. In addition, there are no specific requirements for participation in the training.

The participants in the study were informed about the various survey instruments and gave their consent. The data are stored on an access-restricted cloud and evaluated and analyzed in an anonymized form, complying with the General Data Protection Regulation (GDPR).

### Description of the Intervention: Continuous Education to Become a “Consultant for Digital Healthcare” (“Beratende für Digitale Gesundheitsversorgung”)

In order to enable health care professionals to deal confidently with digital technologies and help shape the digital transformation, the training to become a “Consultant for Digital Healthcare” (“Beratende für digitale Gesundheitsversorgung”) was funded by the Saxony-Anhalt Ministry of Labor, Social Affairs, Health and Equality. The novel training is currently in the pilot and development phase under the direction of the AG Versorgungsforschung | Pflege im Krankenhaus and the Dorothea Erxleben Learning Center of the Medical Faculty of Martin Luther University Halle-Wittenberg. The addressees of the training are health and nursing professionals primarily from the federal state of Saxony-Anhalt. For the pilot phase, approximately 65 participants are registered and went through a modularized curriculum with a workload of 200 teaching units and 5 days of classroom training over a period of 9 months.

On the one hand, the further training is characterized by a strong application orientation that, in addition to theoretical introductions to technical, legal, and financial aspects of digitization, focuses in particular on practical familiarization and experience with new technologies and the promotion of facility-related digitization projects, thus advancing theory-practice transfer and the training of competent multipliers. On the other hand, a special focus is placed on the training of ethical-reflective competencies in order to learn to ethically assess the integration of digital assistance systems into the care process and possible consequences of increasing digitization.

The designed self-study and preparation phase initiates learning content prior to the days of attendance and familiarizes the participants with the first set of content. In the follow-up phase, extensive tasks are designed to ensure sustainable results and learning transfer.

The training is divided into 3 modules (Table 1).

The learning goals listed in Textbox 1 are set to be achieved with the further training program. They were selected based on existing overviews of the necessary acquisition of skills in the area of digital health care [25].

**Table 1:** Overview of the 3 modules in the “Beratende für Digitale Gesundheitsversorgung” training program.

Module	Components
Module I: Fundamentals of digital transformation and its relevance for the nursing professions	Raising awareness and introducing digital health care ePA^a^, DiPA^b^, DiGA^c^,and more Ethical and legal aspects as a prerequisite for digital transformation
Module II: Digital transformation in a value-oriented and professional manner in professional practice	Supply process integration and scenario-based reflection on digital assistive technologies Evidence-based digital transformation—fundamentals of evidence-based work in the context of digital transformation Practical application based on the SEQI^d^ process, including practical support
Module III: Promoting personal development	Basics of project management, moderation methods, creative methods Project work on digital transformation at the facility

^a^ePA: electronic health record.

^b^DiPA: digital nursing applications.

^c^DiGA: digital health applications.

^d^SEQI: Sensitization, Evaluative Introduction, Qualification, Implementation.

**Select learning goals of the continuing education training program.**

- Learners acquire basic knowledge of the digital transformation of health care.
- The learners are sensitized to the need to acquire skills for digital transformation in the context of health care and know its importance for cross-sector care.
- Learners develop a reflective attitude toward digital and assistive technologies.
- The learners know the background and objectives of digital health applications.
- Learners assess the relevance of digital health applications in relation to the professional context.
- Learners can identify, analyze, reflect on, and solve ethical problems by getting to know various ethical theories and models and using practical case studies to demonstrate how to systematically work on and think through an ethical problem.
- Learners know the meaning of ethics in the context of their profession by becoming familiar with the codes, guidelines, and profession-specific guidelines that express this connection.
- Learners can evaluate individual digital assistive technologies (DAT) ethically by getting to know models for the ethical evaluation of DAT.
- Learners can transfer theoretical knowledge into their professional practice by learning the practical use of various technologies and being able to discuss and reflect based on scenarios.
- Learners can critically reflect on the opportunities and risks of using DAT as an additional resource for nursing process planning by testing this with the help of case vignettes.

### Study Population

All participants in the continuing education program for “digital health care consultants” were eligible for inclusion in the study. The target group for participation is fundamentally trained nursing and health care professionals. Since the training is funded by the state of Saxony-Anhalt, participation is also primarily offered to professionals from Saxony-Anhalt. Due to the high demand also from outside Saxony-Anhalt, a quota system was for applicants from other federal states as long as there were free places available.

The inclusion criteria were health professionals (nurse practitioner, health care nurse, nursing specialist, pediatric nurse practitioner, health and pediatric nurse practitioner, geriatric nurse, surgical technologist assistant, anesthesia technician assistant, emergency paramedic, midwife and maternity nurse, physiotherapist, occupational therapist) who are at least 18 years old and have sufficient knowledge of written and spoken German.

The exclusion criteria are a lack of knowledge of the German language and inability to consent.

### Quantitative Study

The “Reflection sheet for recording the competencies of a nurse” questionnaire was designed to record the digital competencies of nursing and health care professionals. Using it, the participants’ self-assessments rate their professional, social-communicative, ethical-reflective, and methodological competencies on a 5-level Likert scale. This takes place as a pre-post comparison, by recording once at the beginning of the training (t1) and once after completion of the training (t3). The questionnaire was completed online and made available via Ilias 1 month before the start of the training. Participants are encouraged to complete the questionnaire as fully as possible. The questionnaire for self-assessment records the subjective feeling of competence by the participants in the areas with corresponding questions for self-assessment (Table 2).

Furthermore, the knowledge levels of the participants were recorded before the start of the modules in order to make possible learning gains visible. This also took place in the sense of a pre-post comparison using single-choice, multiple-choice, open, and closed questions.

**Table 2:** Reflection sheet to record the competencies of the nursing staff in the pre-post assessment.

Category	Items for self-reflection
1. Expertise	Knowledge of DAT^a^ and their classification options Knowledge of the possible uses of DAT Technically correct preparation, implementation, and follow-up of the DAT Recognizing the client's individual care needs Knowledge of how to use DAT Knowledge of SEQI^b^ as a structured implementation model Applying SEQI as a structured implementation model Knowledge of the legal basis of DAT
2. Social-communicative competence	Knowledge of psychological, pedagogical, sociological, and communicative basics within project work Client-appropriate communication and empathy Recognizing and responding to nonverbal signals Carrying out advice, guidance, and training Knowledge of different models of (joint) decision-making Ability to cooperate and work in a team
3. Ethical-reflexive competence	Knowledge of ethical theories and models Knowledge of the importance of ethics in the context of nursing Identification of an ethical problem Analyzing an ethical problem Justifying your own actions ethically Ethical assessment of individual DAT Knowledge of models for the ethical assessment of DAT Application of models for the ethical assessment of DAT Reflected deployment or reflected deployment planning of DAT
4. Methodological competence	Knowledge of scientific research Conducting scientific research Organization and coordination of work processes Presentation techniques Selection and use of assessment instruments

^a^DAT: digital assistive technologies.

^b^SEQI: Sensitization, Evaluative Introduction, Qualification, Implementation.

### Qualitative Study

One series of interviews was planned to qualitatively assess the learning success of the participants and evaluate the overall design of the further training. A total of approximately 10 interviews were planned. The selection of inquiries took place according to the principle of convenience sampling; the achievement of statistical representativeness is not envisaged [37].

By means of the interview guide, the survey was carried out with the clear aim of retrieving specific knowledge necessary to answer the precise and theoretically embedded research question. Accordingly, the interviews conducted after the completion of the training dealt with the following questions or main topics: (1) learning success of the participants (different competencies), (2) participant satisfaction with the course and contents of the training, (3) transfer success as applied in the project work in the facilities.

Thus, the quantitative assessments of the participants in the training are supplemented and explained by the interviews. The methodological recourse to expert interviews for data collection results from the fact that conducting expert interviews already presupposes a good knowledge of the field of investigation, which is guaranteed by the target group. In the expert interviews, the focus was on narrative elements relating to the ideas of a meaningful training concept. The guidelines were developed iteratively using the SPSS method according to [38]. The methodical preparation was developed in the sense of the mechanism-exploring research paradigm of qualitative content analysis according to [39].

### Teaching Analysis Poll (TAP)

TAP is a method for the interim evaluation of teaching units, in which a group discussion takes place with the participants in the absence of the teacher. The evaluation is moderated by uninvolved staff members. The participants first discuss the following 3 questions in small groups: (1) What do you learn most from in this event? (2) What makes your learning difficult? (3) What suggestions for improvement do you have for points perceived as obstructive?

The answers from the groups were discussed in plenary, unclear answers were clarified, and the results were checked for their majority ability. A summary of the majority-acceptable results was sent to the teachers in a feedback meeting, which should take place promptly after the TAP. Ideas are jointly developed on how to implement the participants' suggestions for modifying the content. Subsequently, the teacher discusses with the participants how the training can be modified, if necessary, during the training event and who can contribute to this and how. The goal is to provide feedback for teachers and stimulate reflection and exchange about teaching and learning in the professional development event.

### Open Questions in the Questionnaire Survey

The qualitative part of the quantitative preliminary survey included 2 open-ended questions: (1) What are your expectations for continuing education? (2) What difficulties might arise with regard to continuing education?

### Ethical Considerations

Ethics approval was given for the survey by the Ethics Committee of the Medical Faculty of the Martin Luther University Halle-Wittenberg (processing number: 2023-143 21 06 23).

## Results

Data were collected at the beginning of, in the course of, and at the end of the training program. The program ran from September 2023 until July 2024. The funding of the project started in September 2022. In the course of this period, there were several data points. This study will evaluate the learning outcome of the training program. A change in digital competences in the pre-post comparison is expected. To further investigate the reasons for the expected change, 10 expert interviews were conducted to generate structured feedback from the participants. We expect that the learning objectives of the training will be met. The attendees are expected to increase their level of digital competence in different skills areas: (1) concerning their theoretical knowledge, (2) concerning their hands-on skills planning for the application and practical use of DAT, (3) their reflective skills and applying ethical and legal considerations in their use, (4) applying all that in a structured process of technology implementation within their practical sphere of work. At the time of publication, data analysis and evaluation were underway, and we plan to publish the results in spring 2025.

## Discussion

This pilot study will evaluate a new training program for nurses and allied health services workers to become a “Consultant for Digital Healthcare.” The aim of this study and the appropriate continuing education training program is to educate nursing and health care professionals on the use of DAT. As a result, it is expected that the digital skills of the participants will increase in accordance with the survey instrument used. In addition, the structure and content of the further training will be analyzed and evaluated in a differentiated manner so that targeted adjustments can be made for further runs. This focus gives rise to the following research questions: First, how do continuous education training programs need to be developed, and which focus is appropriate for addressee-appropriate learning goals, course structure, and the general curriculum. Second, which digital competences do the individuals in question require? Third, how should a training program with this specific content and area be evaluated? Fourth, what are the conditions for continued program offering?

In particular, it is expected that the participants will have acquired new knowledge and can apply it in practice. This application focus will be promoted in particular through the project work. The social form of group work and informal exchange between participants is also expected to be crucial for learning success. The special orientation of the continuing education training, with a high level of application relevance, will play a decisive role in preparing the participants for the dynamic and project-related digital transformation of health care [20,21].

This builds on previous research work that initially aimed to assess the needs for digital skills in nursing. A structured training process for the introduction of digital technologies in the facilities was then piloted and evaluated [21]. Ultimately, this preparatory work will be made accessible to a large number of skilled workers in a wide area through further training and converted into a continuing educational program.

The special feature of the training is that it addresses a heterogeneous target group, with many different professional groups from health care, extending from the operational level to the management level. In addition, the aim is to cover as wide a range of technologies as possible in order to provide not only an overview but also a selection of possible technologies for participants’ own practical project.

This study assessing continuing education has some limitations. On the one hand, only methods of subjective self-assessment by the participants are used, which is subject to bias and misjudgment. This point needs to be carefully considered during the evaluation of the results. Furthermore, the pilot phase of the further training program is rather short, which is a necessary prerequisite for further development and a first evaluation of the results and effects of the training on the development of digital competences of the participants. As for the projects within the context of the training program, they are accompanied by the study content and team but need to be developed and implemented with further stakeholders and companies in the real world as a so-called living-lab environment. This implies levels of complexity that cannot be fully controlled by the training program but can still be guided and prepared. The sample size of 65 participants is rather small, but for a first pilot phase, it is enough for the first evaluation and interference regarding appropriate training methods for allied health care workers.

Looking at the results in a broader context, it is important to note that there is still a long way to go to achieve targeted digitally supported health care, for which targeted training and further education are keystones and promoted by the program outlined. General standards for the training should be outlined and accepted on a political level. Furthermore, new technologies must also be developed explicitly for nursing care, with the explicit participation of specialists to address the specific needs of the sector.

## Abbreviations

DAT: digital assistive technologies
GDPR: General Data Protection Regulation
SPSS: collect, check, subsume, and sort
TAP: Teaching Analysis Poll
